# Diseases of the Lungs

**Published:** 1904-05-07

**Authors:** 


(*To be continued.*)

### Diseases of the Lungs.

Pneumonia.—Acute lobar pneumonia is often, even in its earliest stages, an easy condition to diagnose, the difficult cases being mostly those which recur secondarily in the course of some other serious disease. Burt [1] points out the importance of a routine examination of the chest in these conditions, especially when any increase in the respiration rate occurs. The diagnosis from enterica is helped by the discovery of leucocytosis, which is common in pneumonia, but this is not a constant sign. Tuberculous disease may be recognised by a bacteriological examination of the sputum, and in children tuberculous meningitis is generally accompanied by leucopenia. With regard to pleurisy he considers the symptoms usually less pronounced and the pyrexia less uniformly high, while rusty sputum never occurs. Decrease in the chlorides in the urine, moreover, is observed only in pneumonia. Shifting dulness is, when present, an important sign, and so is the enlargement of the affected side of the chest, which, when occurring in pneumonia, is only very slight. Although Burt considers that a needle should not be necessary except to diagnose the nature of the effusion, Lees [2] considers that acute effusion may closely simulate pneumonia. The temperature is no guide, and as to physical signs breath sounds may be audible in childhood over a pleural effusion and inaudible in influenzal pneumonia or the later stages of pneumococcal pneumonia. Cardiac displacement may also occur in pneumonia in children, especially in tuberculous cases. As to treatment Lees lays down the following rules :—Procure sleep (if necessary by morphia) during the first few days. Apply leeches over right heart or venesect when cyanosis occurs or the right auricle dulness is increased. Examine chest daily, putting ice-bags over dull areas as they appear. This checks the growth of the pneumococcus. Give strychnine in adults and belladonna in children, if necessary, hypodermically. Restrict fluids only while right heart is dilated. He also recommends oxygen inhalations, and believes an attack may be cut short by energetic early treatment. Barr[3] concurs that early treatment may abort pneumonia, but says that leeches and venesection are useless, as they can only relieve the systemic circulation ; that ice-bags cannot inhibit the pneumococcal growth, and that daily examination of the chest is meddlesome. Ice-bags to reduce pyæmia should be used on the abdomen. Restriction of fluids he thinks of more use than venesection. He also considers oxygen quite useless. Finlay[4] does not believe that an attack of pneumonia can be cut short. He discountenances digitalis, which has been shown to have no action on the vagus in pyrexial conditions, and is also apt to cause vomiting. Blood-letting, he thinks, may be useful in vigorous subjects, but he doubts the value of opium. Hydrotherapy (cold sponging or baths) he places great reliance on to relieve pain and reduce temperature, and entirely condemns both opium and the antipyretic drugs. Alcohol should only be given when there is a special indication for its use. Elsner,[5] considering the danger of pneumonia to lie in cardiac weakness, induced by toxins in the blood, avoids all depressants and recommends digitalis, strychnine, and adrenalin. Venesection is preferable to nitroglycerin. He advises 15 minims each of sp. æth. co., sp. ammon. aromat., sp. lavand. co., and tinct. valeriani every quarter of an hour. Rectal injections of coffee and whisky, or hypodermics of either sterilised oil or camphor, with saline infusions, may be useful in bad cases.

**Bronchitis and Emphysema**, while sufficiently common conditions, are not for that reason less deserving of careful study. They are particularly conditions affecting persons above middle age. Smith[6] found the average for men 48·6, and for women 46·7 years. After dealing with the morbid anatomy he points out that the conditions of emphysema is essentially a senile change, either local or general, and expresses the opinion that the main stress on the elastic tissue of the lung takes place during expiration. The diagnosis is often easily made, the main point being the exclusion of other causes for the chief symptom—shortness of breath, namely, fluid or solid growths in the chest, blocking of the tubes by secretion, inflammatory consolidation of the lung, and circulatory disturbances. The positive signs are shortness of breath without adequate cause, diminution of cardiac dulness and breath sounds, the shape of the chest, and occasionally hæmoptysis. The treatment lies mainly in preventing catarrh, to which these patients are very liable, and which materially aggravates the condition. Excessive exertion and sudden cooling must be avoided ; damp but not necessarily wet feet and sudden changes in the temperature of the inspired air are dangerous. In the treatment of heart complications he strongly advises venesection for cyanosis and morphia as a cardiac tonic and hypnotic. This drug and also digitalis, which is often useful, should be given in small doses. For bronchitis he believes syrup of Tulu and creosote to be valuable.

[1] Med. Rec., Jan. 2, 1904, p. 14. [2] Brit. Med. Jour., Nov. 28, 1903, p. 1385, and Dec. 5, 1903, p. 1451. [3] Ibid., Dec. 10, 1903, p. 1623. [4] Ibid., Jan. 25, 1904, p. 206. [5] Med. Rec., Jan. 9, 1904, p. 67. [6] Clin. Jour., Nov. 4, 1903, p. 43.

